# Supplementary material for: Adapting an Online Guided Self-Help CBT Programme Targeting Disordered Eating for Students in Aotearoa New Zealand: A Qualitative Study
Source: Nutrients. 2024 Aug 30;16(17):2905. doi: 10.3390/nu16172905 (PMC11396848; doi:10.3390/nu16172905)
Supplement: Supplementary file 1 [file nutrients-16-02905-s001.zip › Document S1- Interview schedule.pdf]

**Accessing Mental Healthcare Questions**

- What might prevent you from getting face-to-face help if you needed it? Or what has prevented you from help seeking in the past?
- What helps you/might help to seek help?
- If you were to seek professional help/when you sought help for a mental health problem, what characteristics would you look for or did you look for in a professional or service? For example, recommendation from a friend, a particular age, gender identity, LGBT+ friendly, etc.
- What are student's views about the way people should deal with disordered eating?
- How do you think other students seek help when they experience disordered eating?
- Do you know of any support available at university for students who are experiencing disordered eating?
- What things would you suggest for mental health professionals and/or services to improve regarding working with students who might be experiencing disordered eating?
- What types of qualities would you look for in an online therapy coach?
- Would you prefer your e-therapy coach to be trained and competent as a mental health professional, or relatable on a personal level?
- What types of things could an online therapy coach do or say to be supportive?
- Are there any other comments you want to make about accessing mental health care?

**Challenges and Strengths**

- What are some of the issues or challenges that people who experience disordered eating face in New Zealand today?
- Are there any other comments you want to make about general issues, difficulties, or strengths you see within your community?

**Intervention Specific Questions**

- What did you think of the programme?
- What did/didn't you like about the programme?
- What did/didn't you like about the personal examples in the programme?
- Do you think the personal examples given are appropriate for people like you?
- What do you think about coach contact? Would you prefer text, phone, unguided, a mix?
- How could we make students aware that this programme is available?
- What changes would you make to the programme?
- What about the programme would make you continue using it?
- What would make you use a therapy app regularly?
- Any other comments you want to make about the app?
