# Supplementary material for: Adapting an Online Guided Self-Help CBT Programme Targeting Disordered Eating for Students in Aotearoa New Zealand: A Qualitative Study
Source: Nutrients. 2024 Aug 30;16(17):2905. doi: 10.3390/nu16172905 (PMC11396848; doi:10.3390/nu16172905)

Module 1: balanced eating

- Introduction video/learn what the program is about

- What do you hope to get out of the program/questions?

- Personal stories about use of the programme

- Education about the importance of regular eating
    - Personal example of binges

- Education about balanced eating

- Eating disorder quiz

- Plan and tips for regular eating
    - Creating own meal plan and meal tracking (what, where and where you eat, and your mindset at the time) (type of meal, time, place, what was eaten, thoughts, binge or restriction, additional behaviours)
    - Meal planning and tracking examples

- Mindful eating exercise

- Session wrap up

- Personal stories of how disordered eating impacted students' lives

- Summary sheet

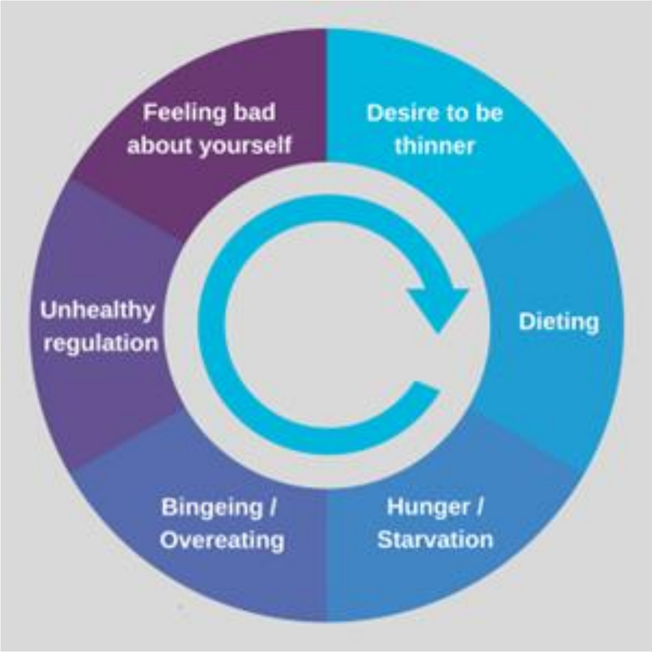

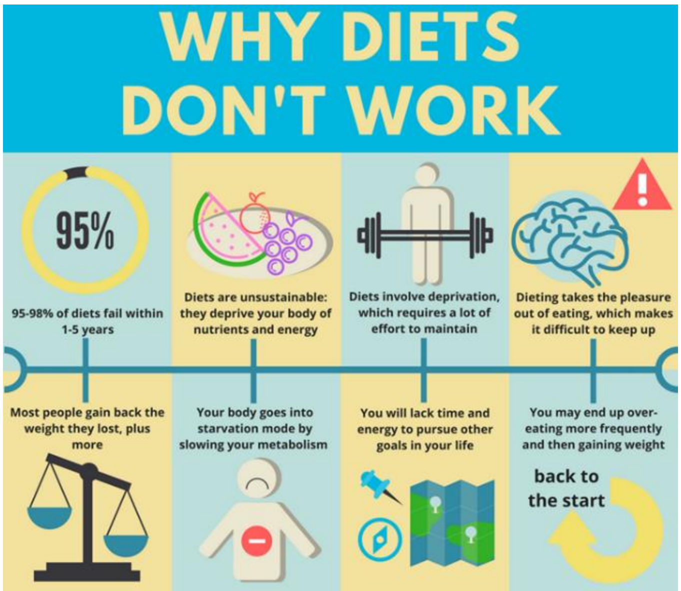

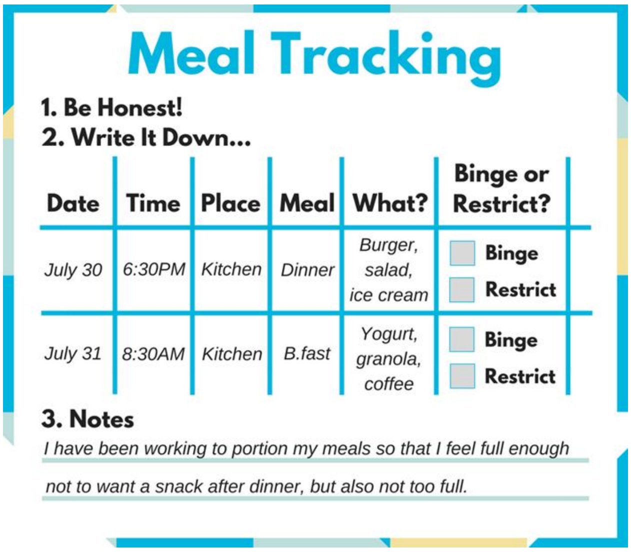

Meal Tracking

1. Be Honest!
2. Write It Down...

Date	Time	Place	Meal	What?	Binge or Restrict?
July 30	*6:30PM*	*Kitchen*	*Dinner*	*Burger, salad, ice cream*	☐ Binge<br>☐ Restrict
July 31	*8:30AM*	*Kitchen*	*B.fast*	*Yogurt, granola, coffee*	☐ Binge<br>☐ Restrict

3. Notes

I have been working to portion my meals so that I feel full enough not to want a snack after dinner, but also not too full.

Module 2: coping well

- Introduction
- Quiz: triggers and coping strategies
- Education about triggers and how they relate to disordered eating behaviour + examples
    - Note down own triggers
- How to deal with triggers
    - Examples + work through own
    - Alternative behaviours to binge eating
    - Personal stories about triggers
- Mindfulness exercise for staying in the present
- Summary sheet

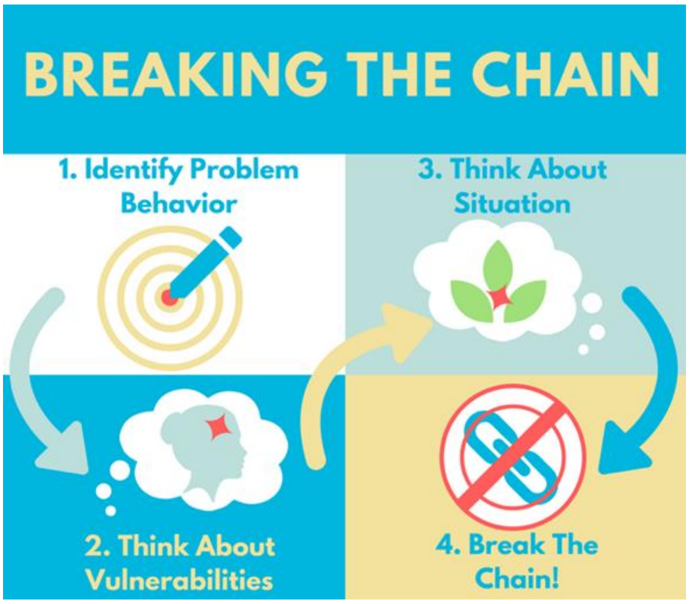

Module 3: thinking well

- Introduction

- Quiz: thinking errors

- Education on negative self-talk and positive self-talk

- Taming the critic: automatic thoughts/thinking errors
    - Brainstorm automatic thoughts
    - Examples of thinking errors

- Evaluate the automatic thought
    - Examples
    - Try it out

- Identifying positive statements about yourself

- Education about the thin body ideal
    - Examples of challenging the thin body ideal
    - Try yourself to challenge the thin body ideal
    - Making a pledge to challenge the thin body ideal

- Education about social comparison
    - Reflect on times you have engaged in social comparison and the consequences that have come from this
    - Tips to help you decrease social comparison
        - Self-monitoring logs
        - Challenging automatic thoughts
        - Behavioural experiments

- Personal stories

- Mindful breathing exercise

- Summary sheet

Module 4: body wellness

- Introduction
- Body wellness quiz
- Education about body image
- Reflection about time spent thinking about body image + thought experiment
- Getting in touch with core values
- Education about shape checking and body avoidance
- Tips for reducing shape checking and body avoidance
- Assessing exercise habits
- Education about consequences of excessive exercise
- Creating a new workout schedule + distinction between healthy and excessive exercise
    - Tips for a health exercise plan + common exercise difficulties
- Personal stories
- Summary sheet

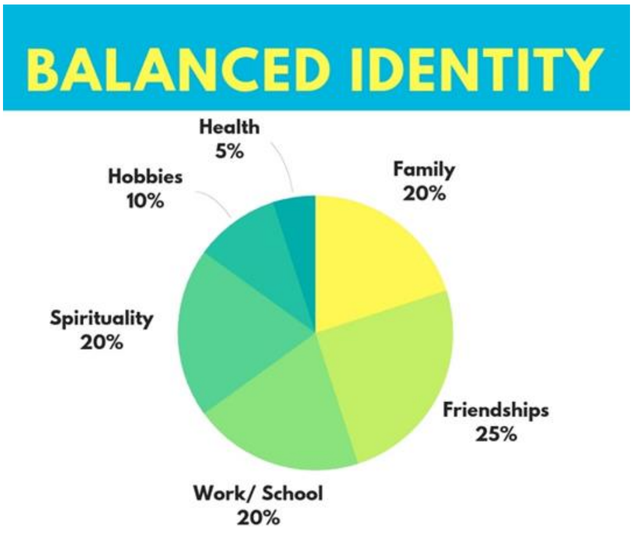

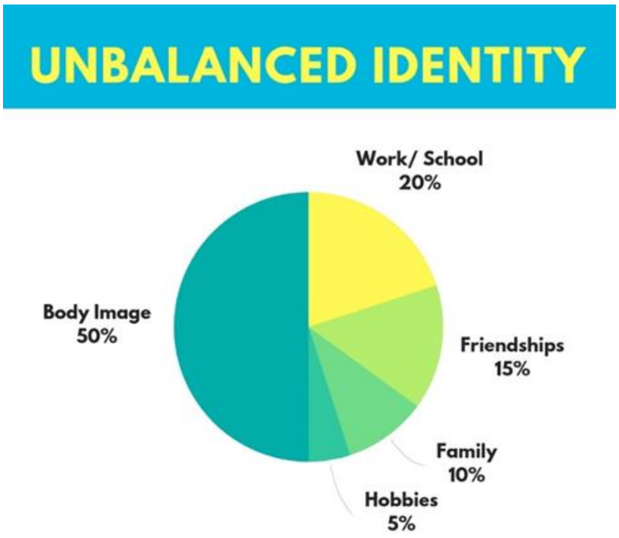

<u>Module 5: media wellness</u>

- Introduction
- Media quiz questions
- Media savvy information
    - Media and cultural awareness
    - Media tricks
    - Impact of picture perfect bias
    - Social media triggers
    - Social media trends
- Reflection of accounts you follow on social media
- Education about the media and social comparison
    - Reflection about own social comparison to media
- Body ideal changing over time + images
- Different beauty practices across cultures
- The idealized body
    - Men
    - Women
    - Health risks
- Social media trend: body transformation
- Challenges
    - Post photo on social media that captures something you like about yourself with no filters or no retouching
    - Post a favourite quote from a role model on social media
- Personal stories
- Summary sheet

Module 6: relationship wellness and relapse prevention

- Introduction

- Importance of supportive relationships
  - Reflect on when a relationship has triggered you to feel badly which then triggered disordered eating behaviour and thoughts

- Tips for successful relationships

- Break ups

- Communication exercise + examples + practice

- Coping with criticism; tips + practice

- Criticism; body talk
  - Personal stories
  - How to confront a friend, romantic partner or family member

- Reflection
  - How do you currently deal with criticism?

- Maintain your gains
  - What are you proud of
  - Be prepared
  - Reflect
  - Support
  - What if you slip
  - Personal stories

- Summary sheet

**The Event**	*A situation, relationship, or practical problem that's concerning you. Explain what you're talking about. Clarify what exactly about the situation is causing your concern.*
**Your Emotions**	*How do you feel in this situation? Explain how the situation affects your emotions. Everyone can argue about opinions – but not about emotions. Expressing your emotions clearly can prevent a lot of confusion.*
**Your Needs**	*Remember, the other person cannot read your mind, so you need to clearly express your needs.*
**The Consequences**	*How will the changes improve the situation for yourself and the other person? Explain the positive consequences that will arise when your needs are met. Be concrete about it.*

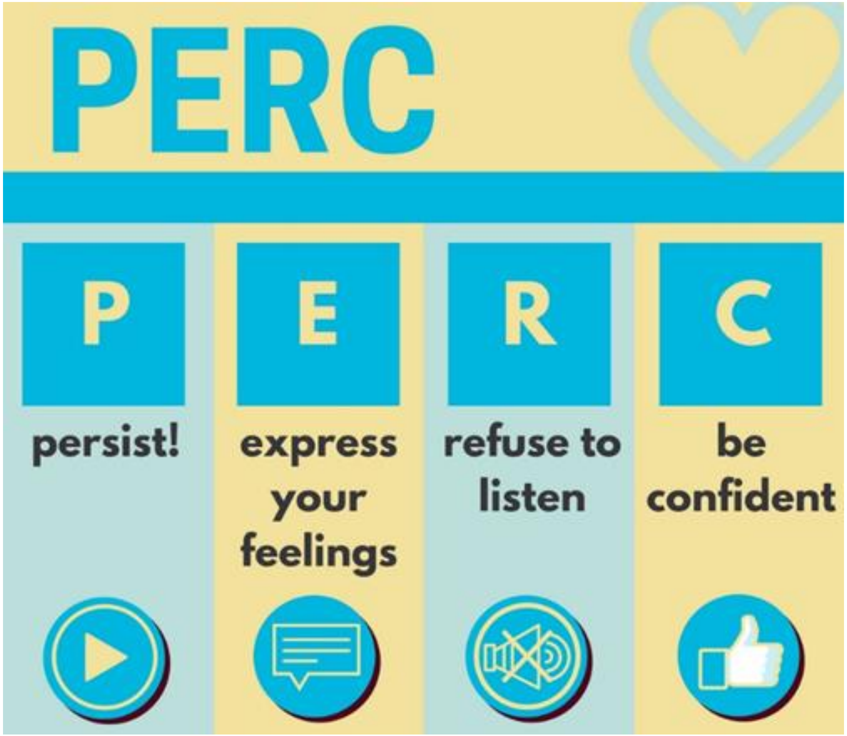

Supplement: Supplementary file 1 [file nutrients-16-02905-s001.zip › Document S2 Summary of modules.pdf]
