# Supplementary material for: Adapting an Online Guided Self-Help CBT Programme Targeting Disordered Eating for Students in Aotearoa New Zealand: A Qualitative Study
Source: Nutrients. 2024 Aug 30;16(17):2905. doi: 10.3390/nu16172905 (PMC11396848; doi:10.3390/nu16172905)
Supplement: Supplementary file 1 [file nutrients-16-02905-s001.zip › Figure S1 Complete code template which demonstrates the heirarchy of themes.pdf]

1. **Programme content**
    1.1. ***Content is relevant, relatable and acceptable***
        1.1.1. But make sure it sounds genuine
            1.1.1.1. *Important to be mindful about how personal stories are used*
                1.1.1.1.1. Risk of creating comparison
                1.1.1.1.2. Wording needs to suit the Aotearoa/New Zealand context

    1.2. ***Diverse needs and customization***
        1.2.1. The social media challenge is not for everyone
            1.2.1.1. *Not everyone uses social media this way*
            1.2.1.2. *The dangers of social media*
            1.2.1.3. *Alternative challenge*
        1.2.2. Challenging but not 'triggering'
        1.2.3. Logbooks and food diaries might not be suitable for some
            1.2.3.1. *Support resources*
        1.2.4. Providing a choice for coach communication
            1.2.4.1. *Level of formality*
            1.2.4.2. *Stage of their journey*
        1.2.5. It's about more than the thin ideal
            1.2.5.1. *Body positivity*
            1.2.5.2. *Masculine ideal*

    1.3. ***Addressing important issues***
        1.3.1. The cost of living and food anxiety
        1.3.2. Peer influences
            1.3.2.1. *'How can I support my peers'*

2. **Engagement and retaining users**
    2.1. ***Facilitators***
        2.1.1. Ability to see and monitor progress
        2.1.2. Coach responsivity
            2.1.2.1. *Sensitivity*
            2.1.2.2. *Follow-up*
        2.1.3. Notifications

    2.2. ***Barriers***
        2.2.1. The student lifestyle
            2.2.1.1. *Module length*
                2.2.1.1.1. Feeling bored
        2.2.2. It's hard to accept it as a problem
            2.2.2.1. *Potential to promote awareness*
            2.2.2.2. *Trialling the programme*

    2.3. ***Marketing of online intervention***
        2.3.1. Easy accessibility
        2.3.2. Social media
        2.3.3. Around the university
            2.3.3.1. *Student Association*

3. **Ethics and management of personal information**

3.1. Confidentiality and privacy

3.2. Safety and training

3.2.1. The spectrum from relatable to professional

3.2.1.1. The need to be able to relate
