# Supplementary material for: Adapting an Online Guided Self-Help CBT Programme Targeting Disordered Eating for Students in Aotearoa New Zealand: A Qualitative Study
Source: Nutrients. 2024 Aug 30;16(17):2905. doi: 10.3390/nu16172905 (PMC11396848; doi:10.3390/nu16172905)
Supplement: Supplementary file 1 [file nutrients-16-02905-s001.zip › Table S1- Proposed change to the Aotearoa New Zealand version of everyBody..pdf]

Area	Changes/recommendations	Comments
Language		
	Use language more suitable to cultural context.	E.g. ‘first year’ instead of ‘freshman year’.
	Make audio recordings for mindfulness activities with New Zealand accent.	
Content		
	Reduce the number of personal stories included.	Include them where they seem natural and appropriate.
	Add in reference to diverse body ideals.	Reflects current and cultural body image ideal trends.
	Remove media challenge.	High participant consensus that it would not be useful. Media challenge is not a key component of Cognitive Behavioural Therapy (CBT).
	Included a weekly symptoms tracker, and space at the end of each module for participant to reflect on their progress.	Most participants discussed progress monitoring as a key to retaining engagement in the programme.
	Include an introduction module. This will involve a phone/video call between participant and coach.	This phone call will provide the space to create a point of connection between the coach and intervention user. Within this phone call, the coach should be empathetic to the distress the individual is experiencing and show a genuine interest to help [59]. Lastly, this phone call will allow for the intervention user to ask any questions or state any concerns they have with going forward in the intervention. Together, this preliminary module is likely to create some intervention buy-in before the individual is faced with the potentially distressing task of addressing their eating habits.
	Coach to provide on the rationale of meal tracking to send to participants.	Wilson and Vitousek (1999) [59] discuss several recommendations that can facilitate adherence to self-monitoring. Careful explanation of the rationale for self-monitoring will be important to consider. The coach can send this out as further educational resources for participants who are having difficulty with engaging in the food self-monitoring.

	Break down modules into several sessions that take 10-15 minutes to complete.	There was high consensus among the participants that the modules should be short in length.
Coaching		
	Pre-screen for coach communication preference (telephone, email, text, chat).	Participants demonstrated various preferences for coach contact.
Notifications		
	Reminder notification once per week.	Discuss with participant when the best time is to send out this notification to increase the likelihood that it is a meaningful reminder.
Promotion		
	Involve university student services.	The participants in the current study highlighted that student-led services were seen as a trusted place that many students seek help for a variety of problems. It would be useful to collaborate with students and student organisations and co-design help-seeking initiatives to promote the engagement and uptake of everyBody.
